# Deaths from Fall-Related Traumatic Brain Injury — United States, 2008–2017

**DOI:** 10.15585/mmwr.mm6909a2

**Published:** 2020-03-06

**Authors:** Alexis B. Peterson, Scott R. Kegler

**Affiliations:** 1 Division of Injury Prevention; National Center for Injury Prevention and Control; CDC.

One in 10 U.S. residents aged ≥18 years reports falling each year (*1*). Among all age groups, falls can cause serious injury and are the second leading cause of traumatic brain injury (TBI)–related deaths (*2*). TBI is a head injury caused by a bump, blow, or jolt to the head or body or a penetrating head injury that results in disruption of normal brain function.* CDC estimated national and state-specific rates and trends for TBI-related deaths (TBI deaths) caused by unintentional falls (fall-related TBI deaths) among U.S. residents during 2008–2017, by selected decedent characteristics. The national age-adjusted rate of fall-related TBI deaths increased by 17% from 2008 to 2017. Rate trends at the national level increased significantly for nearly all decedent characteristics, with the most notable increases observed among persons living in noncore (i.e., most rural), nonmetropolitan counties and those aged ≥75 years. Analysis of state-specific rate trends determined that rates of fall-related TBI deaths increased significantly in 29 states over the 10-year study period. A fall can happen to anyone of any age, but falls are preventable. Health care providers and the public need to be aware of evidence-based strategies to prevent falls, given that rates of fall-related TBI deaths are increasing. Health care providers can educate patients on fall and TBI prevention, assess their risk for falls, and when needed, encourage participation in appropriate evidence-based fall prevention programs.^†^

National Vital Statistics System multiple-cause-of-death database on death certificates filed in 50 states and the District of Columbia (DC) were analyzed to determine the incidence of fall-related TBI deaths among U.S residents by year, decedent characteristics (sex, age group, race/ethnicity, and urban/rural residence classification status^§^), and state of residence. To identify cases, an initial screen for *International Classification of Diseases, Tenth Revision* (ICD-10) underlying-cause-of-death codes in the range W00–W19 was performed, indicating an unintentional fall as the underlying cause of death. A fall-related death was further identified as a TBI death when any of the ICD-10 multiple-cause-of-death codes indicated a TBI-related diagnosis (*2*).^¶^ Study years 2008–2017 were selected to support estimation of 10-year national and state-specific trends.

Annual death rates and accompanying 95% confidence intervals (CIs) were calculated per 100,000 population by integrating the National Vital Statistics System data with U.S. bridged-race population estimates.** With the exception of age-group rates, death rates were age-adjusted to the U.S. year 2000 standard age distribution. National and state-specific rate trends of fall-related TBI deaths were modeled using Joinpoint regression software (version 4.6.0.0; National Cancer Institute) to estimate average annual percent changes (AAPCs) for the 10-year study period. AAPCs were considered statistically significant at α = 0.05.

During 2008–2017, the national age-adjusted rate of fall-related TBI deaths increased by 17%, from 3.86 per 100,000 persons to 4.52 (Table 1), representing 17,408 fall-related TBI deaths in 2017. State-specific age-adjusted rates ranged from 2.25 (Alabama) to 9.09 (South Dakota) during 2017 (Figure). Considering only the study endpoint years (2008 and 2017), the number of fall-related TBI deaths increased in 49 of 51 jurisdictions (50 states and DC), and corresponding age-adjusted rates increased in 45 of these 49 jurisdictions (Supplementary Table, https://stacks.cdc.gov/view/cdc/85245). The largest AAPCs in rates of fall-related TBI deaths occurred in Maine (6.5%), South Dakota (6.1%), and Oklahoma (5.2%). A significant increase in rates occurred in 29 states (Arkansas, California, Colorado, Connecticut, Florida, Indiana, Iowa, Kansas, Louisiana, Maine, Maryland, Massachusetts, Minnesota, Missouri, Nebraska, Nevada, New Hampshire, North Carolina, Ohio, Oklahoma, Oregon, Pennsylvania, Rhode Island, South Carolina, South Dakota, Tennessee, Texas, Virginia, and Wisconsin). The remaining 21 states and DC experienced no significant change in rates.

**TABLE 1:** Number* and rate^†^ of traumatic brain injury–related deaths caused by unintentional falls — United States, 2008–2017^§^

Year	No. of deaths	Rate (95% CI)
2008	12,311	3.86 (3.80–3.93)
2009	12,804	3.94 (3.87–4.01)
2010	13,386	4.05 (3.98–4.12)
2011	13,632	4.02 (3.95–4.09)
2012	14,272	4.12 (4.05–4.19)
2013	15,064	4.26 (4.19–4.33)
2014	15,918	4.40 (4.33–4.47)
2015	16,258	4.42 (4.35–4.49)
2016	16,694	4.44 (4.37–4.51)
2017	17,408	4.52 (4.45–4.59)

**Abbreviation:** CI = confidence interval.

* Numbers exclude decedents with unknown age.

^†^ Deaths per 100,000 population, age-adjusted to the 2000 U.S. standard population; decedents with unknown age were excluded.

^§^ Based on multiple-cause-of-death data from the National Center for Health Statistics (NCHS) Vital Statistics System (https://www.cdc.gov/nchs/nvss/deaths.htm) and NCHS Bridged-Race Population data (https://www.cdc.gov/nchs/nvss/bridged_race.htm).

**FIGURE:**
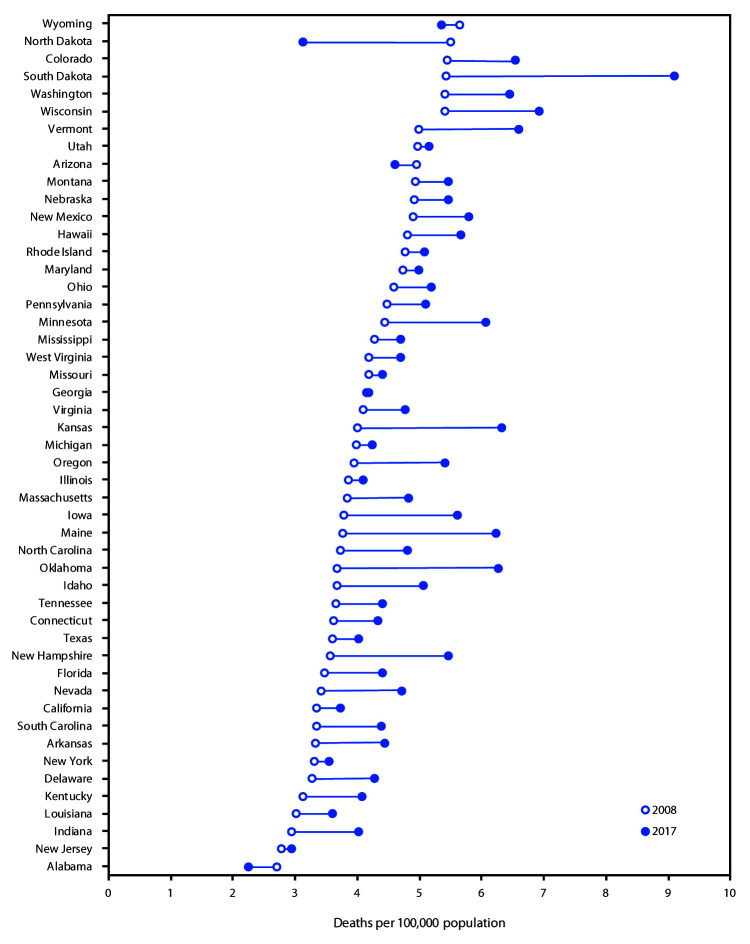
Age-adjusted* rate of traumatic brain injury–related deaths caused by unintentional falls, by state^†^ — United States, 2008 and 2017 * Age-adjusted to the 2000 U.S. standard population. ^†^ Forty-nine states; Alaska and the District of Columbia not shown because total case count was <20.

During 2017, national rates of fall-related TBI death were highest among persons aged ≥75 years (54.08 per 100,000) and males (6.31) (Table 2). Notably, the rate among persons aged ≥75 years was approximately eight times higher than that among those aged 55–74 years (6.24), and the rate among males was nearly double that of females (3.17). For the period 2008 to 2017, significantly increasing rate trends in fall-related TBI deaths were identified for both males and females, persons aged ≥55 years, non-Hispanic whites, non-Hispanic blacks, and Hispanics, and across all levels of urbanization. The largest modeled rate increases occurred among persons living in noncore nonmetropolitan counties (AAPC = 2.9%) and those aged ≥75 years (AAPC = 2.6%). The only significantly decreasing national rate trend identified was for persons aged 0–17 years (AAPC = −4.3%).

**TABLE 2:** Numbers* and rates^†^ of traumatic brain injury–related deaths caused by unintentional falls, by decedent characteristics — United States, 2008 and 2017^§^

Characteristic	2008		2017		2008–2017 rate trend AAPC (95% CI)
	No. of deaths	Rate (95% CI)	No. of deaths	Rate (95% CI)	
**Total**	**12,311**	**3.86 (3.80 to 3.93)**	**17,408**	**4.52 (4.45 to 4.59)**	**1.8^¶^ (1.5 to 2.1)**
**Sex**					
Male	7,129	5.49 (5.36 to 5.62)	10,180	6.31 (6.19 to 6.44)	1.6^¶^ (1.3 to 2.0)
Female	5,182	2.69 (2.61 to 2.76)	7,228	3.17 (3.09 to 3.24)	1.9^¶^ (1.5 to 2.4)
Age group (yrs)**					
0–17	75	0.10 (0.08 to 0.12)	54	0.07 (0.05 to 0.09)	−4.3^¶^ (−7.6 to −0.9)
18–34	304	0.43 (0.38 to 0.48)	295	0.39 (0.34 to 0.43)	−1.1 (−3.0 to 0.8)
35–54	1,241	1.43 (1.35 to 1.51)	1,137	1.37 (1.29 to 1.45)	−0.3 (−1.2 to 0.5)
55–74	2,855	5.22 (5.03 to 5.41)	4,470	6.24 (6.05 to 6.42)	1.8^¶^ (1.4 to 2.3)
≥75	7,836	42.89 (41.94 to 43.83)	11,452	54.08 (53.09 to 55.07)	2.6^¶^ (2.0 to 3.2)
**Race/Ethnicity^††^**					
White	10,501	4.09 (4.01 to 4.17)	14,472	4.90 (4.82 to 4.98)	2.1^¶^ (1.7 to 2.4)
Black	581	1.99 (1.82 to 2.16)	844	2.29 (2.13 to 2.45)	1.6^¶^ (0.2 to 3.1)
AI/AN	68	4.13 (3.08 to 5.18)	121	5.16 (4.20 to 6.11)	1.0 (−1.9 to 4.1)
A/PI	361	3.61 (3.22 to 3.99)	645	3.68 (3.39 to 3.97)	0.3 (−0.7 to 1.3)
Hispanic	777	3.23 (2.98 to 3.48)	1,282	3.51 (3.31 to 3.71)	1.2^¶^ (0.3 to 2.0)
Not stated	23	NA^§§^	44	NA^§§^	NA^§§^
**Level of urbanization**					
Large central metro	3,320	3.77 (3.64 to 3.90)	4,604	4.31 (4.18 to 4.44)	1.4^¶^ (1.2 to 1.6)
Large fringe metro	2,946	3.90 (3.76 to 4.05)	4,051	4.31 (4.17 to 4.44)	1.4^¶^ (0.5 to 2.3)
Medium metro	2,673	3.96 (3.81 to 4.11)	3,889	4.72 (4.57 to 4.87)	2.1^¶^ (1.5 to 2.7)
Small metro	1,181	3.76 (3.54 to 3.97)	1,791	4.76 (4.54 to 4.98)	2.2^¶^ (1.4 to 3.1)
Micropolitan (nonmetro)	1,292	4.10 (3.87 to 4.33)	1,793	4.98 (4.75 to 5.22)	2.1^¶^ (1.5 to 2.8)
Noncore (nonmetro)	899	3.65 (3.41 to 3.89)	1,280	4.60 (4.34 to 4.86)	2.9^¶^ (2.5 to 3.4)

**Abbreviations:** AAPC = average annual percent change; AI/AN = American Indian/Alaska Native; A/PI = Asian or other Pacific Islander; CI = confidence interval; NA = not available.

* Numbers exclude decedents with unknown age.

^†^ Per 100,000 population, age-adjusted to the 2000 U.S. standard population; rates exclude decedents with unknown age.

^§^ Based on multiple-cause-of-death data from the National Center for Health Statistics (NCHS) Vital Statistics System (https://www.cdc.gov/nchs/nvss/deaths.htm) and NCHS Bridged-Race Population data (https://www.cdc.gov/nchs/nvss/bridged_race.htm).

^¶^ Statistically significant at α = 0.05.

** Age group rates are not age-adjusted.

^††^ Whites, blacks, AI/ANs, and A/PIs were non-Hispanic; Hispanics could be of any race.

^§§^ Accompanying rates are not available because of lack of corresponding population denominator data.

## Discussion

Nationally, nearly 17,500 fall-related TBI deaths occurred during 2017, and state-specific age-adjusted rates ranged from 2.25 (Alabama) to 9.09 (South Dakota). The rate of this health event significantly increased during 2008–2017 in 29 states, and the national rate increased by 17%. This increase in the national rate of fall-related TBI deaths is consistent with findings from a recent CDC surveillance report that estimated a 22% increase in this health event during 2006–2014.^††^

Variations in the rate of fall-related TBI deaths among states might have partially resulted from urban and rural differences in the risk of traumatic injury mortality (*3*). U.S. rural regions experience a higher rate of TBI-related mortality (*4*), and heterogeneity in the availability and accessibility of resources (e.g., access to high-level trauma centers and rehabilitative services) can result in disparities in post-injury outcomes (*5*). Over the 10-year study period, noncore, nonmetropolitan counties experienced the most rapidly increasing rates. These results are consistent with previous findings of higher TBI-related mortality rates among nonmetropolitan counties compared with those in metropolitan counties across the United States (*4*).

During 2017, the rate of fall-related TBI deaths was higher among males; this finding might result from circumstances of the falls, such as a higher proportion of men falling from heights (e.g., ladders) (*6*) leading to moderate or severe injuries, including a TBI. The highest rate of fall-related TBI deaths in 2017 was among adults aged ≥75 years, and over the study period, this group experienced the largest increase in rates among all age groups, consistent with older age being a major risk factor for falls (*7*). CDC’s Stopping Elderly Accidents, Deaths, & Injuries (STEADI)^§§^ initiative can aid health care providers in screening older patients for risk for falls, assessing modifiable risk factors, and intervening to reduce risk using effective interventions. Health care providers might consider prescribing exercises that incorporate balance, strength and gait activities, such as tai chi, and reviewing and managing medications linked to falls (*8*). Actions the public can take to prevent falls include talking to their health care provider about their or their parents’ risk for falls, performing strength and balance exercises, having an annual eye exam, and making the home safer (e.g., removing tripping hazards).

The findings in this report are subject to at least three limitations. First, estimated annual rates and trends in rates of fall-related TBI deaths might be affected by misclassification or incomplete reporting of the cause of death on death certificates, which could lead to overestimation or underestimation of this health event (*9*). Second, misclassification of race and ethnicity on death certificates is a common occurrence, particularly for American Indian/Alaska Native, Asian/Pacific Islander, and Hispanic populations and could lead to an underestimation of deaths among these populations (*10*). Finally, in cases of multiple trauma, non-TBI diagnoses might have also contributed to deaths included in the analysis.

A fall can happen to anyone of any age and can cause serious injuries, including a TBI. Although falls are preventable, the public should be aware that fall-related TBI deaths are increasing in many states as well as nationally. Nationally, this increase might be explained by longer survival following the onset of common diseases such as stroke, cancer, and heart disease^¶¶^ or be attributable to the increasing population of older adults*** in the United States. In older adults, evidence-based fall prevention strategies can prevent falls and avert costly medical expenditures (*8*). Additional research is needed to determine the magnitude of medically treated falls that could be prevented and direct medical costs that could be averted by employing evidence-based fall prevention strategies in other age groups. Nonetheless, annual wellness visits might serve as a time to focus on previously assessed risk factors for falls and to update personalized prevention plans.

**Summary**

**What is already known about this topic?**

Falls can cause serious injuries, including a traumatic brain injury (TBI). Unintentional falls represent the second leading cause of TBI-related death.

**What is added by this report?**

The national age-adjusted rate of fall-related TBI deaths increased by 17% from 2008 to 2017; rates increased significantly in 29 states and among nearly all groups, most notably persons living in noncore nonmetropolitan counties and those aged ≥75 years.

**What are the implications for public health practice?**

Health care providers can educate patients about falls and TBIs, assess fall risk, and encourage participation in evidence-based fall prevention programs. Annual wellness visits might serve as a time to review previously assessed fall risk factors and update personalized prevention plans.

## Footnotes

* https://www.cdc.gov/traumaticbraininjury/index.html.

† https://www.cdc.gov/homeandrecreationalsafety/pdf/falls/cdc_falls_compendium-2015-a.pdf.

§ https://www.cdc.gov/nchs/data/series/sr_02/sr02_166.pdf.

¶ TBI injury-related diagnosis codes identified by ICD-10 include S01.0–S01.5, S01.7–S01.9, S02.0–S02.1, S02.3, S02.7–S02.9, S04.0, S06.0–S06.9, S07.0–S07.1, S07.8–S07.9, S09.7–S09.9, T90.1–T90.2, T90.4–T90.5, T90.8–T90.9.

** https://www.cdc.gov/nchs/nvss/bridged_race.htm.

†† https://www.cdc.gov/traumaticbraininjury/data/tbi-deaths.html.

§§ https://www.cdc.gov/steadi/.

¶¶ https://www.sciencedirect.com/science/article/pii/S002243750600051X?via%3Dihub.

*** https://www.census.gov/prod/2014pubs/p25-1140.pdf.
